# Bioinformatic analysis identifies LPL as a critical gene in diabetic kidney disease via lipoprotein metabolism

**DOI:** 10.3389/fendo.2025.1620032

**Published:** 2025-07-17

**Authors:** Qian Dong, Huan Xu, Pengjie Xu, Jiang Liu, Zhouji Shen

**Affiliations:** Department of Nephrology, The Affiliated Lihuili Hospital of Ningbo University, Ningbo, China

**Keywords:** diabetic kidney disease, lipoprotein lipase, immune cell infiltration, lipid metabolism, bioinformatic

## Abstract

**Background:**

Diabetic kidney disease (DKD) is a common and serious complication of diabetes, affecting approximately 40% of patients with the condition. The pathogenesis of DKD is complex, involving multiple processes such as metabolism, inflammation, and fibrosis. Given its increasing incidence and associated mortality, there is an urgent need to identify novel pathogenic genes and therapeutic targets.

**Methods:**

This study systematically identified hub DKD-associated genes and their potential molecular mechanisms through bioinformatic analysis. Gene expression datasets from DKD patients and healthy controls were obtained from the GEO database. Hub genes were screened using differential expression analysis, weighted gene co-expression network analysis (WGCNA), LASSO regression, random forest (RF) algorithms, and consensus clustering for DKD patient classification. Additionally, immune cell infiltration analysis was performed on differentially expressed genes to explore the relationship between hub genes and the immune microenvironment. Potential drugs targeting LPL were predicted based on gene-drug interaction analysis. Immunohistochemistry was used to verify the expression of LPL and TNF-α in kidney tissues from patients with varying degrees of DKD severity, as well as their relationship with kidney function impairment.

**Results:**

This study revealed that LPL, a lipoprotein metabolism gene, plays a crucial role in DKD, participating in cholesterol and glycerolipid metabolism as well as PPAR signaling. LPL expression was negatively correlated with pro-inflammatory M1 macrophages and various subsets of T cells, including naïve CD4 T cells and gamma delta T cells, while positively correlated with follicular helper T cells, suggesting its immune-regulation effects in DKD progression. Potential LPL-targeting drugs, such as Ibrolipim, anabolic steroid, and acarbose, might mitigate DKD. LPL expression was decreased with DKD severity and was correlated with TNF-α and kidney dysfunction markers, indicating its key role in DKD progression.

**Conclusion:**

LPL is a pivotal regulator of lipid metabolism and immune inflammation in DKD. Potential drugs targeting LPL offer new candidates for precision treatment of DKD. These findings lay a theoretical foundation for understanding the molecular mechanisms of DKD and developing LPL-based therapeutic strategies.

## Introduction

Diabetic kidney disease (DKD) is a common and serious microvascular complication of diabetes and is one of the leading causes of chronic kidney disease (CKD) and end-stage renal disease (ESRD), affecting approximately 40% of patients with diabetes (1, 2). The early stages of DKD are often asymptomatic, resulting in delayed diagnosis in huge amount of DKD patients. This delayed detection contributes to the global rise in DKD prevalence and mortality rates, especially in low- and middle-income countries, making it a significant public health burden (3–5). The pathophysiology of DKD is complex, involving the interplay of multiple factors such as metabolic disturbances induced by hyperglycemia, hemodynamic abnormalities, oxidative stress, chronic inflammation, and genetic susceptibility (6–9).

Hyperglycemia activates several key biochemical pathways such as the polyol pathway, the advanced glycation end product (AGE) pathway, and protein kinase C, which further exacerbate kidney damage (10, 11). Concurrently, glomerular hyperfiltration and overactivation of the renin-angiotensin system (RAS) lead to increased intraglomerular pressure, promoting mesangial matrix expansion and podocyte apoptosis (12, 13). Besides, Hyperglycemia promotes the secretion of pro-inflammatory cytokines including C-reactive protein (CRP), interleukin-6 (IL-6), and tumor necrosis factor-alpha (TNF-α), all of which have been shown to contribute to DKD progression and cardiovascular disease development (14, 15). Oxidative stress and inflammatory responses further exacerbate cellular damage, while tubular epithelial cell trans differentiation and extracellular matrix deposition drive fibrotic progression (6, 16, 17). Additionally, genetic background and epigenetic regulation influence disease susceptibility and progression (18). These mechanisms collectively contribute to glomerulosclerosis, tubulointerstitial fibrosis, and progressive renal function decline. Given the limitations of current treatment strategies, identifying new pathogenic factors and therapeutic targets, particularly through in-depth exploration of molecular mechanisms, may offer new opportunities to improve the diagnosis and treatment of DKD (19).

In this study, a comprehensive analysis to explore key pathogenic genes and potential therapeutic targets of DKD was conducted. Gene expression datasets from DKD patients and healthy controls were obtained from the Gene Expression Omnibus (GEO) database and subjected to multi-level bioinformatic analysis. Differential expression analysis including Kyoto Encyclopedia of Genes and Genomes (KEGG), Gene Ontology (GO) enrichment, gene set enrichment analysis (GSEA) and weighted gene co-expression network analysis (WGCNA) in combination with LASSO regression and random forest (RF) algorithms to identify hub genes, and simultaneously classified DKD patients using consensus clustering. Immune cell infiltration analysis was performed to elucidate the role of inflammatory response in the progression of DKD and to provide theoretical support for immunotherapy. In addition, potential targeted drugs and drug-gene interactions involving hub genes were conducted. The bioinformatic conclusion was approved by immunohistochemistry. This study highlights the role of lipid metabolism disorders and their associated immune-inflammatory responses, providing new insights and potential targets for the early diagnosis, classification and targeted treatment of DKD.

## Materials and methods

### Data search and download

In this study, datasets related to DKD were obtained from the GEO database (http://www.ncbi.nlm.nih.gov/geo) for subsequent data analyses. Both the GSE30122 (training set) and GSE104948 (validation set) datasets are based on microarray technology. The gene expression data for GSE30122 was obtained from the GEO database. These files are typically provided as log2-transformed and pre-normalized expression matrices by the data submitters or GEO processing pipeline. Following this, our preprocessing involved handling probes that correspond to multiple genes by averaging their expression levels to represent a single gene symbol. Subsequently, Z-score normalization was applied to the log2-transformed data to standardize gene expression distributions across samples, which facilitates comparability in downstream analyses. The GSE30122 dataset was selected as the training set, which contained the gene expression profiles of 19 DKD patients and 50 healthy controls. We utilized GSE104948 dataset was used as a validation set to verify the robustness of the model and the reliability of the GSE104948-GPL22945 subset, which included gene expression profiles of 7 DKD patients and 18 healthy controls, directly relevant to diabetic kidney disease.

### Identification of differentially expressed genes

The “Limma” package (version 3.60.5) in R was utilized to screen the differentially expressed genes (DEGs) between DKD patients and healthy controls in the training set (GSE30122). Genes with a *P* < 0.05 and |log2(FC)| > 0.585 were considered significantly differentially expressed. This threshold, corresponding to a fold change of 1.5, was selected to balance statistical significance with the retention of genes exhibiting subtle yet potentially critical biological changes, thereby ensuring a comprehensive yet reliable set of differentially expressed genes for downstream functional analyses. The expression profiles of DEGs were visualized using a volcano plot and a heatmap generated with the pheatmap package (version 1.0.12) and the ggplot2 package (version 3.5.1), respectively.

### Enrichment analysis

To better understand the biological functions of key genes, GO functional enrichment analysis and KEGG pathway enrichment analysis were performed on the identified DEGs using the clusterProfiler package (version 4.12.6) in R. GO enrichment analysis evaluated gene enrichment across three domains: Biological Processes (BP), Molecular Functions (MF), and Cellular Components (CC), offering a comprehensive understanding of their functional characteristics.

Additionally, GSEA was conducted to examine the distribution of DEGs within known functional pathways. By analyzing the ranking of gene expression, GSEA determines whether specific gene sets are significantly enriched in particular biological pathways. In this study, GSEA was performed using the clusterProfiler package (version 4.12.6), with the Hallmark gene sets from the Molecular Signatures Database (MSigDB) as the reference standard. *P*<0.05 was considered statistically significant.

### WGCNA analysis of differentially expressed genes

To further identify the key gene modules associated with DKD, this study used the R package WGCNA (version 1.72-5) to construct a gene co-expression network. WGCNA is a widely used bioinformatic method that can reveal co-expression patterns between genes and identify gene modules associated with specific biological characteristics (20).

First, the pickSoftThreshold function was used to select an optimal soft threshold to ensure the sparsity of the network and to generate a robust co-expression network. Subsequently, gene co-expression was assessed by calculating the Pearson correlation coefficient between gene pairs in the adjacency matrix. These weighted co-expression relationships enabled the evaluation of interaction strength between genes and the identification of key gene modules within the network. Finally, genes from key modules associated with DKD were extracted for further analysis and validation.

### LASSO and RF analysis

To further identify hub genes closely related to DKD, the key module genes screened by WGCNA were subjected to Least Absolute Shrinkage and Selection Operator (LASSO) regression analysis and RF analysis.

In the LASSO regression analysis, we used the R package glmnet (version 4.1-8) and 5-fold cross-validation to determine the optimal regularization parameter. By incorporating L1 regularization, the number of variables in the model was controlled by compressing less important coefficients to zero, thereby reducing model complexity and retaining the most representative gene features.

The RF analysis, an ensemble learning method based on decision trees, assesses the importance of each gene by constructing multiple decision trees. RF analysis was performed using the R package randomForest (version 4.7-1.1), and the most critical genes were identified based on their importance scores, measured as Mean Decrease Gini.

The results of LASSO regression and RF analysis were analyzed through Venn diagram intersection analysis using EVenn (http://www.ehbio.com/test/venn/#/) to screen out hub genes related to DKD for subsequent in-depth research.

### Consensus cluster analysis

To explore molecular heterogeneity within the DKD patient cohort based on the five identified hub genes (LPL, BCAM, SERPINE2, GCNT3, and CTNNBIP1), consensus clustering was performed on the DKD samples from the training dataset (GSE30122). The analysis was conducted using the ConsensusClusterPlus R package (v1.58.0) applying the k-means algorithm with Euclidean distance. Cluster stability was evaluated for k values ranging from 2 to 9 (maxK=9), using 1000 resampling iterations (reps=1000) with 80% samples randomly selected in each iteration (pItem=0.8). The optimal number of clusters was determined based on the consensus cumulative distribution function (CDF) plots and the stability of consensus matrices. DKD samples were subsequently classified into the optimal number of subtypes, and the expression patterns of the five hub genes across these subtypes were visualized using heatmaps and box plots.

### Immune cell infiltration assessment

To evaluate the composition of 22 types of immune cells in the samples, the CIBERSORT algorithm was used to conduct immune cell infiltration analysis based on differentially expressed genes. A box plot was utilized to visually present the differences in immune cell composition between the two groups. In addition, Pearson correlation analysis was conducted to assess the relationships between hub genes and various immune cells. The R package corrplot was used to generate a heatmap of the correlation between genes and immune cells, aiming to visualize the association between hub genes and immune infiltration characteristics.

### Gene-drug prediction

To explore potential drug targets of hub genes, this study leveraged the Drug-Gene Interaction Database (DGIdb, https://www.dgidb.org/) to perform drug association analysis on the screened hub genes to identify possible drug-gene interactions. The interaction scores provided by DGIdb are quantitative measures derived from integrated information across various public databases and literature sources, reflecting the strength of potential drug-gene associations. Our study primarily utilized these pre-computed scores for predictive analysis.

### Clinical sample collection and immunohistochemical analysis

To verify the correlation between LPL and the severity of DKD and its renal function indicators, 20 renal tissue samples were collected, including 10 paraffin-embedded renal biopsy samples from patients with varying severities of DKD and 10 healthy control samples from non-tumor renal tissue adjacent to renal tumors. The age and gender of the samples in each group were matched to eliminate the influence of confounding factors. All sample collections were carried out with informed consent from patients and approved by the hospital’s ethics committee (KY2024SL295-01).

The collected kidney tissue samples were fixed in 4% paraformaldehyde buffer at room temperature for 24 hours, then embedded in paraffin and sectioned into 4 μm thick sections. The sections were hydrated, and nonspecific binding was blocked with 5% goat serum for 1 hour at room temperature. The sections were then incubated overnight at 4°C with primary antibodies, including anti-LPL antibody (1:200, RayBiotech, # 144-64237) and anti-TNF-α antibody (1:200, Abcam, #ab1793). The next day, the sections were incubated with biotinylated secondary antibodies (1:500, Ray Biotech, # 144-00277) at room temperature for 1 hour. Subsequently, 3,3’-diaminobenzidine (DAB, MilliporeSigma) was applied for 5 minutes for staining, followed by hematoxylin counterstaining at room temperature for 60 seconds. After counterstaining, the sections were dehydrated with gradient ethanol, cleared with xylene, and mounted with neutral balsam. The completed sections were observed and recorded under an optical microscope (Olympus, Tokyo, Japan) to evaluate the expression level of LPL protein in DKD and control tissues.

## Results

### Differentially expressed gene identification

**Figure 1:**
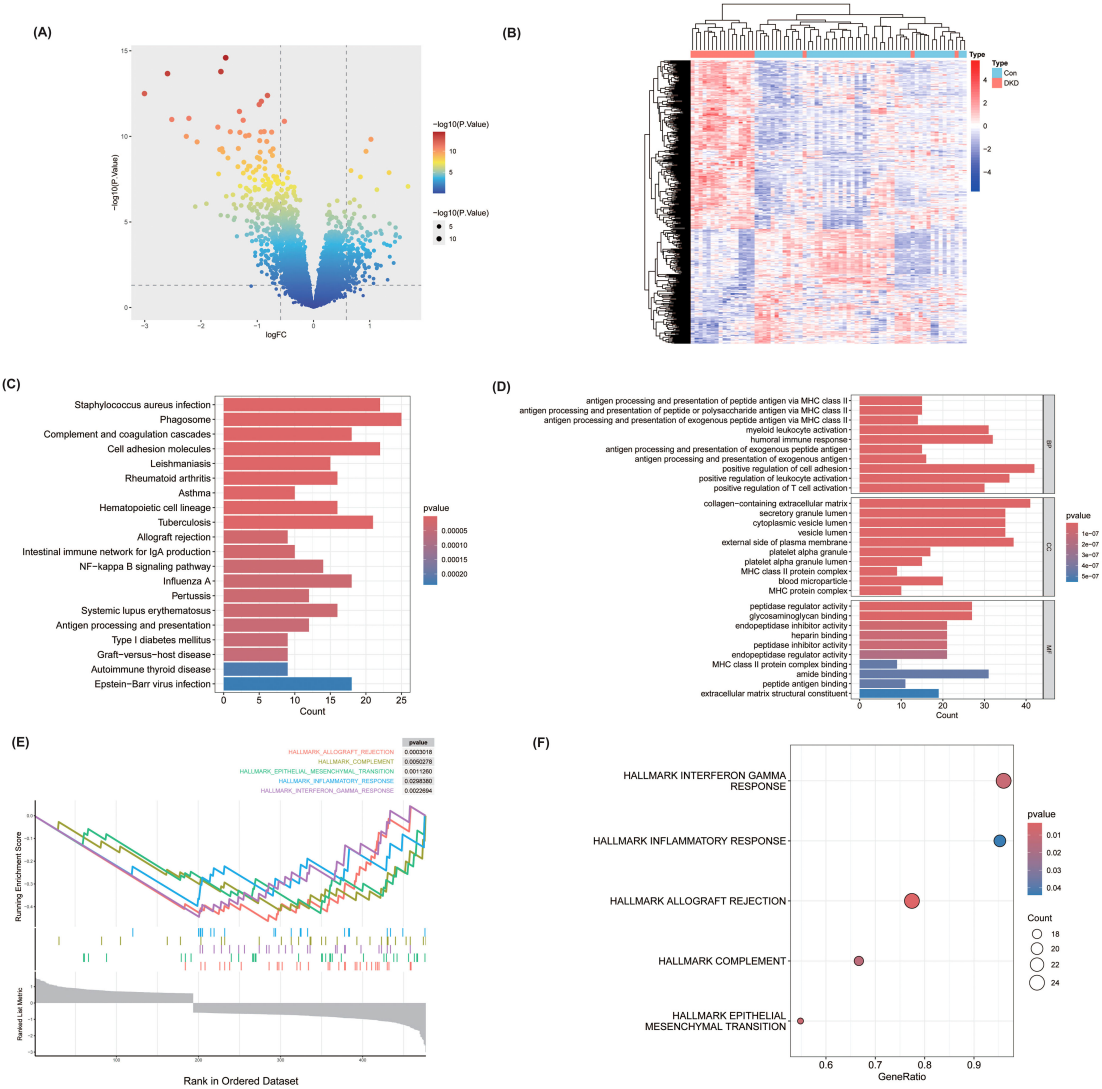
Differentially expressed genes (DEGs) analysis and enrichment results in DKD patients versus healthy controls. **(A)** Volcano plot of DEGs identified in the GSE30122 dataset based on the criteria |log2FC| > 0.585 and P < 0.05 in diabetic kidney disease (DKD) patients compared to controls. **(B)** Heatmap showing the expression levels of selected DEGs in DKD and control groups. **(C)** KEGG pathway enrichment analysis of DEGs. **(D)** GO enrichment analysis illustrating DEGs significantly associated with biological processes (BP), molecular functions (MF), and cellular components (CC). **(E, F)** Gene Set Enrichment Analysis (GSEA) of DEGs.

Through analyses of the GSE30122 dataset from the GEO database, a total of 477 differentially expressed genes (DEGs) were identified between DKD patients and healthy controls, based on the criteria of P < 0.05 and |log2FC| > 0.585. Among these, 193 genes were upregulated, and 284 were downregulated (**Figures 1A, B**).

To further explore the BP in which these DEGs may be involved in, we conducted GO and KEGG enrichment analyses. The KEGG analysis revealed that these genes were significantly enriched in immune and inflammation-related pathways, such as the NF-kappa B signaling pathway, Antigen processing and presentation, and Phagosome (**Figure 1C**). In GO analysis, differentially expressed genes were significantly enriched in BP, MF, and CC, involving key processes such as positive regulation of cell adhesion, collagen-containing extracellular matrix, and MHC class II protein complex binding (**Figure 1D**). Additionally, the GSEA analysis further confirmed significant enrichment of these DEGs in pathways such as Interferon gamma response, inflammatory response, epithelial mesenchymal transition, complement, and allograft rejection (**Figures 1E, F**), providing further insights into the molecular mechanisms underlying DKD.

### WGCNA to identify key modules

**Figure 2:**
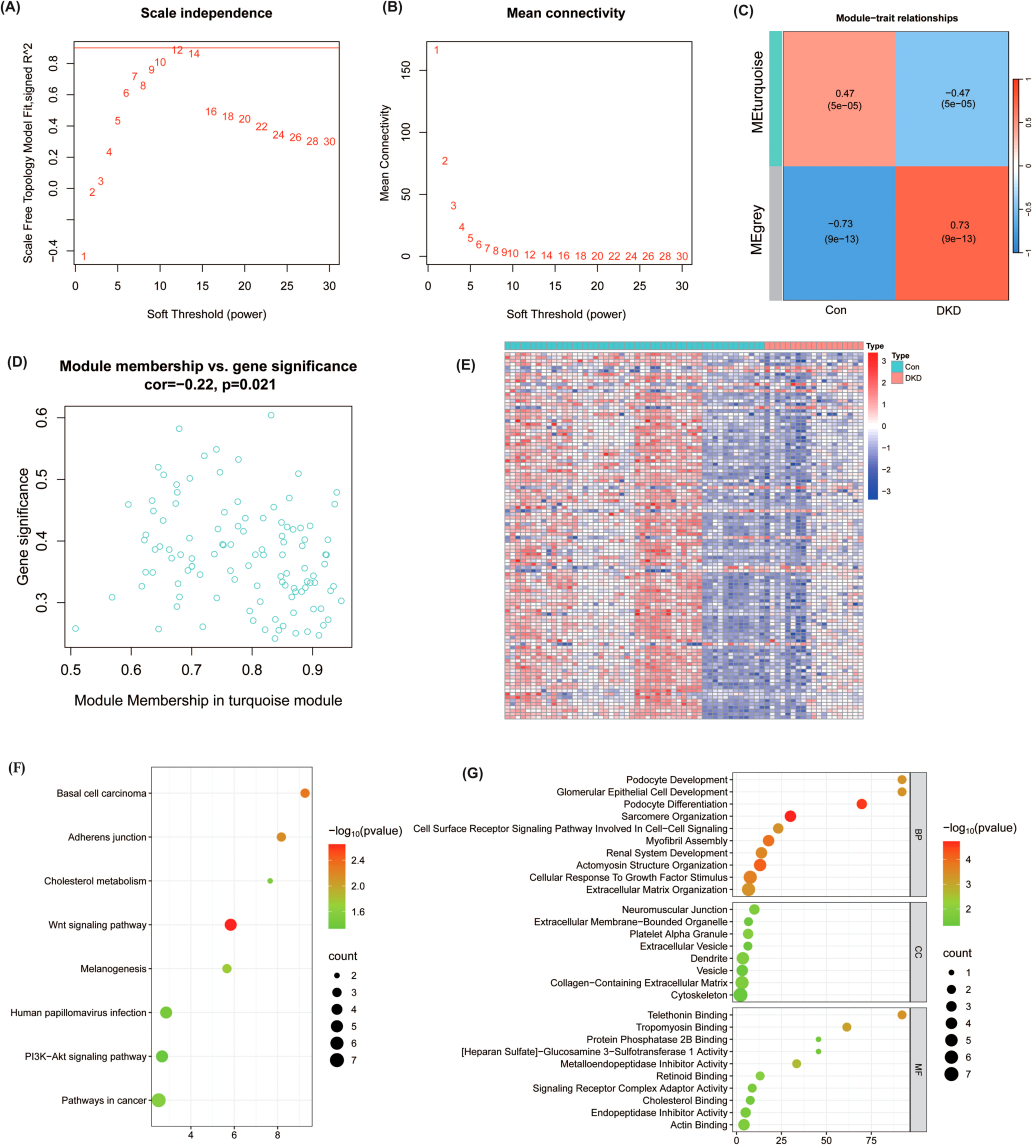
Weighted Gene Co-expression Network Analysis (WGCNA) of DEGs in DKD patients and control samples. **(A, B)** Scale-free topology model fit index as a function of the soft-thresholding power. A soft-thresholding power of 12 was chosen to ensure scale-free topology in the network construction. **(C)** Module-trait relationships showing a significant association between the turquoise module and DKD (r = 0.47, P = 5e-05). **(D)** Scatter plot of module membership versus gene significance for genes in the turquoise module, with a notable correlation (r = -0.22, P = 0.021). **(E)** Heatmap depicting the expression levels of genes within the turquoise module across DKD and control samples. **(F, G)** Functional enrichment analysis of genes in the turquoise module via KEGG and GO analysis.

To identify gene modules associated with DKD, WGCNA was performed on 477 differentially expressed genes from 19 DKD samples and 50 normal samples. Based on the scale-free topological network construction (**Figures 2A, B**), the soft threshold was set to 12. Hierarchical clustering identified multiple modules containing different numbers of co-expressed genes, and the similarity analysis of module feature genes revealed the correlation between modules. Correlation analysis showed that the MEturquoise module was significantly correlated with DKD (r = 0.47, P = 5e-05) (**Figure 2C**). Within the MEturquoise module, 110 genes were found to be correlated with DKD (r = -0.22, P = 0.021, **Figure 2D**). The expression levels of genes in the MEturquoise module are shown in **Figure 2E**. To further investigate the function of the genes in the MEturquoise module, KEGG and GO analyses were conducted. The results revealed significant associations with pathways such as the Wnt signaling pathway, adherens junction, and cholesterol binding (**Figures 2F, G**). These findings further suggest that the genes within the MEturquoise module and the associated pathways may play a key role in the progression of DKD, offering potential directions for its diagnosis and therapy.

### LASSO and RF analysis to identify hub genes

**Figure 3:**
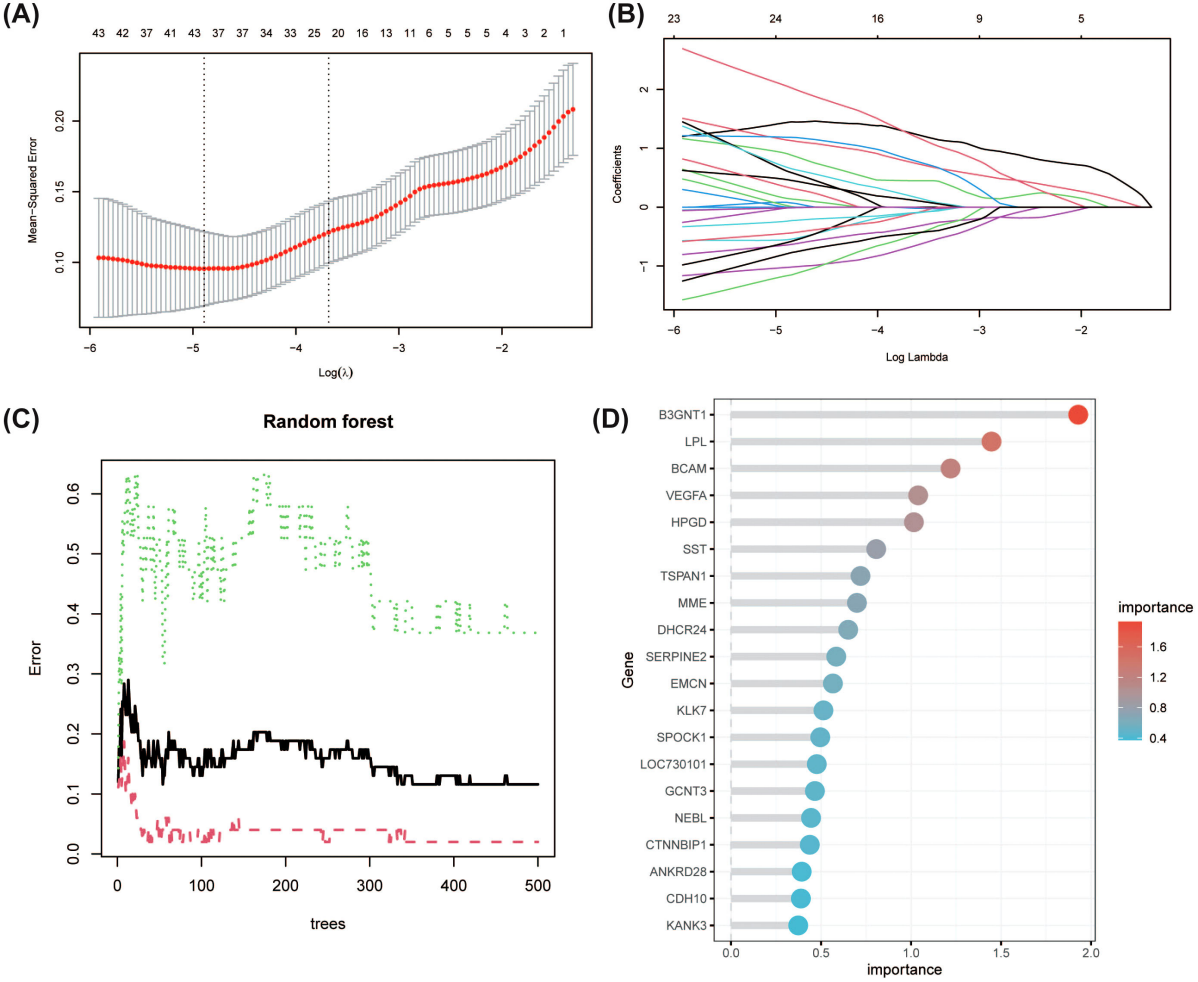
Identification of key predictive genes using LASSO regression and Random Forest (RF) analysis. **(A)** LASSO regression model with 5-fold cross-validation to select the optimal regularization parameter (λ). The minimum mean squared error was used to determine the optimal λ value, resulting in the selection of key predictive genes. **(B)** LASSO coefficient profiles for each gene as a function of the regularization parameter λ. Genes with non-zero coefficients at the optimal λ were identified as key genes. **(C)** RF analysis showing the error rate as a function of the number of decision trees. **(D)** Top 20 genes ranked by importance score (Mean Decrease Gini) in the RF analysis.

LASSO regression and RF analysis were performed on the key module genes screened by WGCNA to identify key genes with significant predictive value. After LASSO regression determined the optimal regularization parameter through 5-fold cross-validation, 24 key genes were finally identified (**Figures 3A, B**). In the RF analysis, the top 20 genes were screened out based on the gene importance score (Mean Decrease Gini) (**Figures 3C, D**).

**Figure 4:**
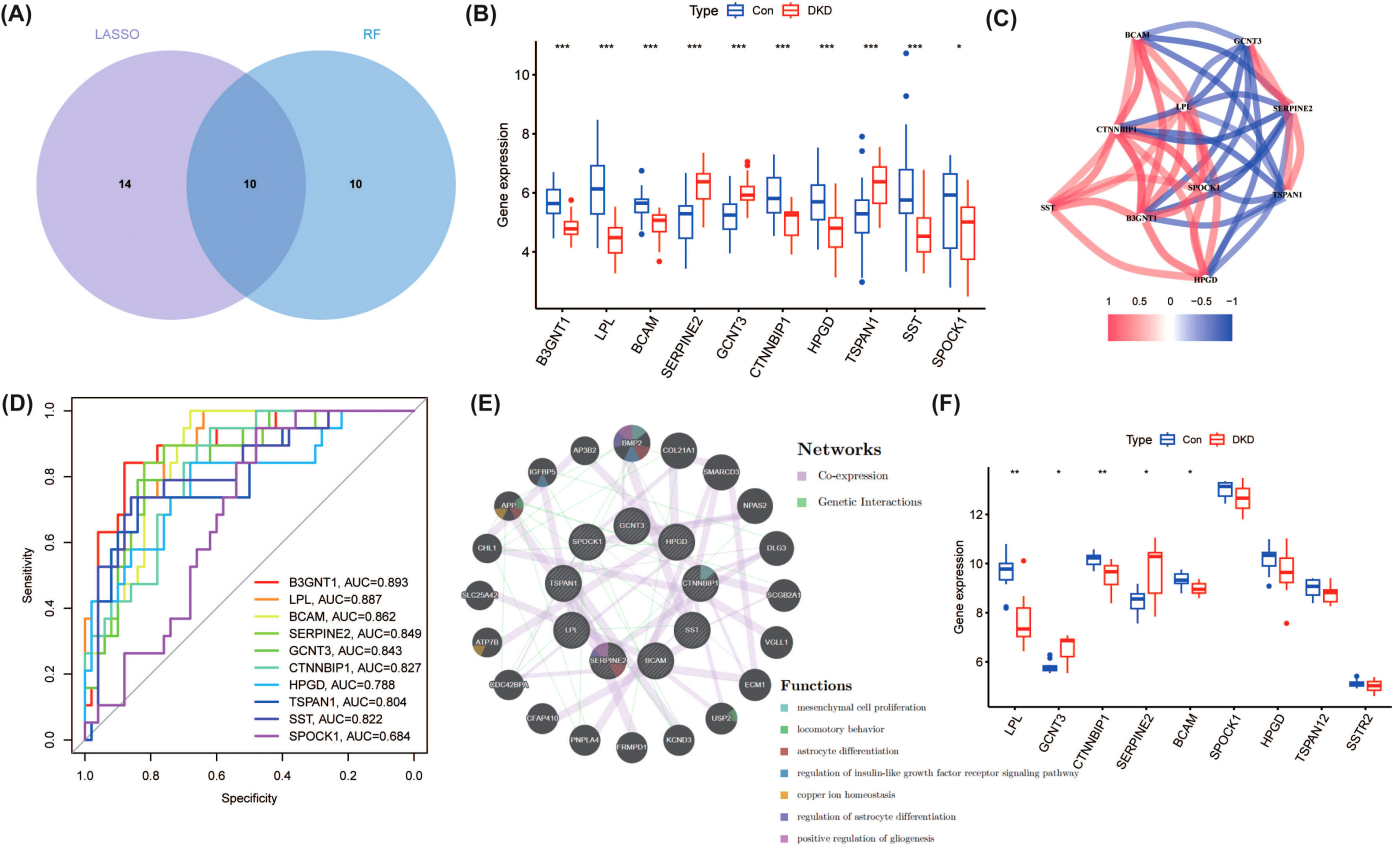
Integrated analysis of hub genes identified by LASSO and RF models in DKD. **(A)** Venn diagram depicting 10 overlapping genes identified by both LASSO and RF analyses. **(B)** Expression levels of the overlapping genes in DKD and control samples. *P<0.05, ***P<0.001. **(C)** Correlation analysis of the expression levels of the 10 overlapping genes. **(D)** Receiver Operating Characteristic (ROC) analysis for each gene. **(E)** Gene-gene interaction network generated via the GENEMANIA database. **(F)** Validation (GSE104948) of gene expression in an independent dataset identified five hub genes (LPL, BCAM, SERPINE2, GCNT3, and CTNNBIP1) closely associated with DKD. *P<0.05, **P<0.01.

A total of 10 overlapping genes were identified from both LASSO and RF analysis, which were visualized using Venn diagrams (**Figure 4A****).** The expression levels of these genes are shown in **Figure 4B**. Further correlation analysis revealed the correlations between the expression levels of these genes (**Figure 4C**). To evaluate the potential of these genes in diagnosis, ROC analysis was performed, and the results showed that the combination of these genes had good predictive performance (**Figure 4D**). In addition, the gene-gene interaction network constructed using the GENEMANIA database revealed that the functions of these genes are closely related to mesenchymal cell proliferation, locomotory behavior, astrocyte differentiation, and regulation of insulin-like growth factor receptor signaling (**Figure 4E**). Finally, the expression levels of these genes were verified on the validation set, and 5 hub genes closely related to DKD (LPL, BCAM, SERPINE2, GCNT3, and CTNNBIP1) were screened out (**Figure 4F**), providing important candidate targets for further functional research and drug development.

### Consensus cluster analysis

**Figure 5:**
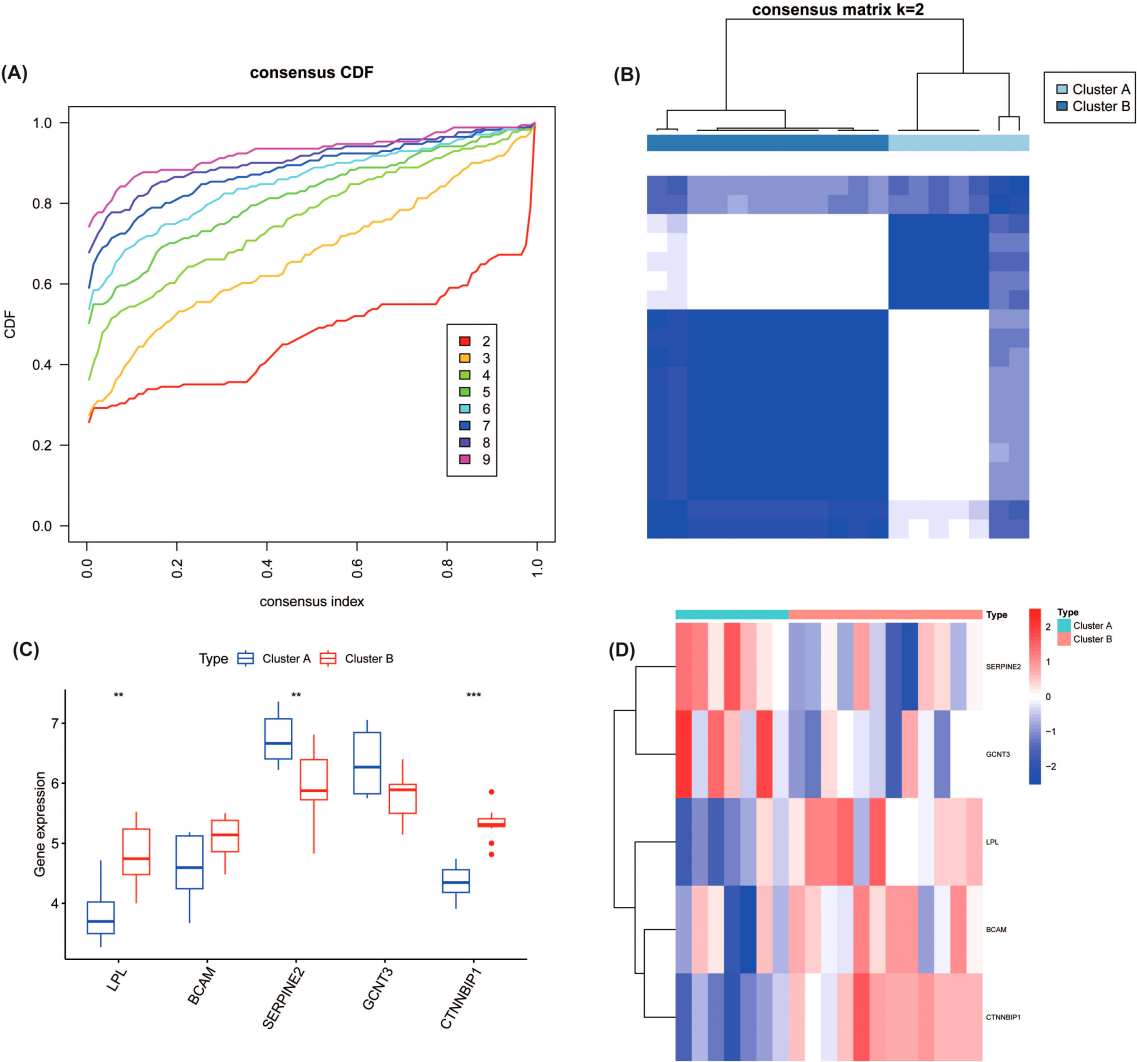
Consensus clustering analysis of five hub genes in DKD patients. **(A)** Cumulative Distribution Function (CDF) curve for consensus clustering with different values of k. **(B)** Consensus matrix for clustering, showing optimal stability at k=2, resulting in two molecular subtypes (Cluster A and Cluster B) based on the expression of hub genes. **(C)** Expression levels of the hub genes (LPL, BCAM, SERPINE2, GCNT3, and CTNNBIP1) in the two identified subtypes. ***P<0.001. **(D)** Heatmap of gene expression between Cluster A and Cluster B **P<0.01.

Consensus cluster analysis was performed on the DKD patient samples from the training set using the expression profiles of the five hub genes (LPL, BCAM, SERPINE2, GCNT3, and CTNNBIP1) to identify distinct molecular subtypes. Evaluation of the consensus CDF curves and the consensus matrix heatmaps indicated that k=2 yielded the most stable clustering solution (**Figures 5A, B**). Consequently, the DKD samples were classified into two robust molecular subtypes, designated Cluster A and Cluster B.

There were significant differences in the expression levels of core genes between the two subtypes (**Figures 5C, D**). Specifically, compared to Cluster B, Cluster A exhibited significantly reduced expression of LPL (P < 0.01) and CTNNBIP1 (p < 0.001), along with lower expression of BCAM. In contrast, Cluster A showed significantly elevated expression of SERPINE2 (p < 0.01) and higher expression of GCNT3.

### Immune cell infiltration assessment

**Figure 6:**
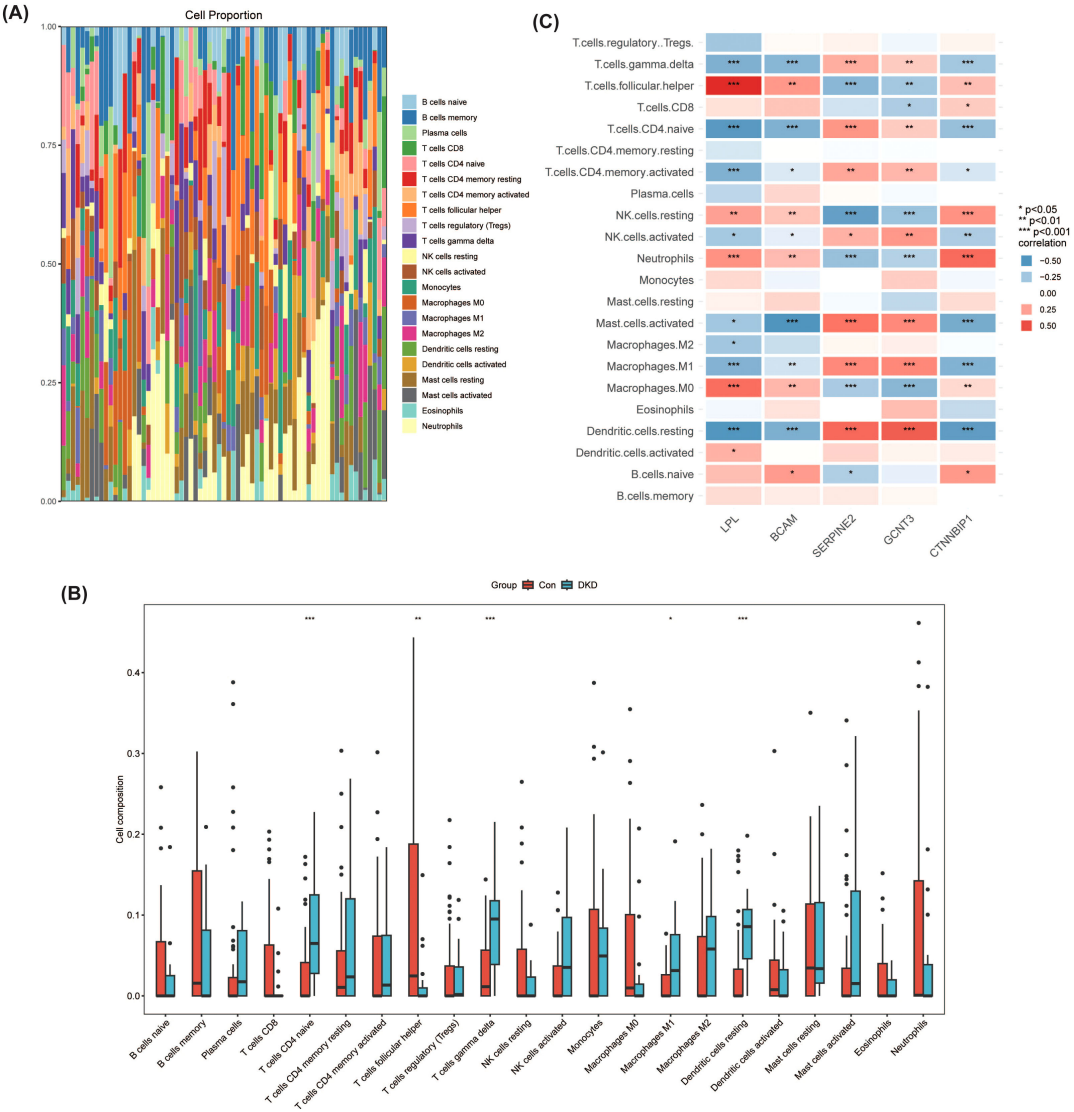
Analysis of immune cell composition and hub gene immune associations in DKD using CIBERSORT. **(A)** Proportional distribution of immune cell types in DKD patients and healthy controls (Control group). **(B)** Comparative analysis of immune cell infiltration in DKD and control groups. *P<0.05, **P<0.01, ***P<0.001. **(C)** Correlation analysis of hub genes (LPL, BCAM, SERPINE2, GCNT3, and CTNNBIP1) with various immune cell types. *P<0.05, **P<0.01, ***P<0.001.

To evaluate the immune microenvironment, the proportions of 22 types of immune cells in DKD patient and healthy control samples were estimated using the CIBERSORT algorithm based on the overall gene expression profiles from the training dataset. The analysis revealed distinct immune cell composition profiles between the groups (**Figure 6A**). Comparing the estimated immune cell proportions between the DKD group and the healthy control group, significant alterations in several immune cell subsets were observed (**Figure 6B**). Notably, DKD patients exhibited significantly increased proportions of M1 macrophages, naïve CD4 T cells, and gamma delta T cells compared to controls, suggesting a shift towards a pro-inflammatory immune microenvironment in DKD.

To further explore the potential relationship between the key genes identified in this study and the immune landscape, we performed a correlation analysis specifically between the expression levels of the five identified hub genes (LPL, BCAM, SERPINE2, GCNT3, and CTNNBIP1) and the estimated proportions of various immune cell types (**Figure 6C**). This analysis revealed that LPL expression was positively correlated with M0 macrophages and follicular helper T cells, while being negatively correlated with M1 and M2 macrophages. These correlations suggest that LPL may play a role within the pro-inflammatory immune context of DKD. Additionally, significant correlations were observed between BCAM and SERPINE2 expression and various immune cells, indicating their potential involvement in modulating inflammatory responses and immune infiltration in DKD.

### Hub gene enrichment analysis

The results showed that LPL was significantly associated with Cholesterol metabolism (P = 0.012), Glycerolipid metabolism (P = 0.015) and PPAR signaling pathway (P = 0.018, Table 1). These findings suggest that LPL may play a crucial role in lipid metabolism disorders, and through regulating lipidmetabolism and inflammatory responses, it could contribute to the progression of DKD.

**Table 1:** Results of hub gene functional enrichment analysis.

Term	P-value	Genes
Mucin type O-glycan biosynthesis	0.009	GCNT3
Cholesterol metabolism	0.012	LPL
Glycerolipid metabolism	0.015	LPL
PPAR signaling pathway	0.018	LPL
Wnt signaling pathway	0.041	CTNNBIP1

Further gene-drug interaction analysis identified a variety of potential targeted drugs that may affect the pathological progression of DKD by regulating the activity of the LPL gene. The analysis results showed that IBROLIPIM had the strongest interaction with LPL (Interaction score = 1.875), suggesting that it has a high potential in regulating LPL activity. Other compounds, such as anabolic steroid (Interaction score=1.250) and ACARBOSE (Interaction score=0.625) also showed significant interaction scores with LPL, indicating that they may have a significant effect on the functional regulation of LPL (**Supplementary Figure 1**, **Supplementary Table 1**). These results offer promising therapeutic targets for DKD intervention.

### Immunohistochemical analysis of the correlation between LPL expression and DKD severity

**Figure 7:**
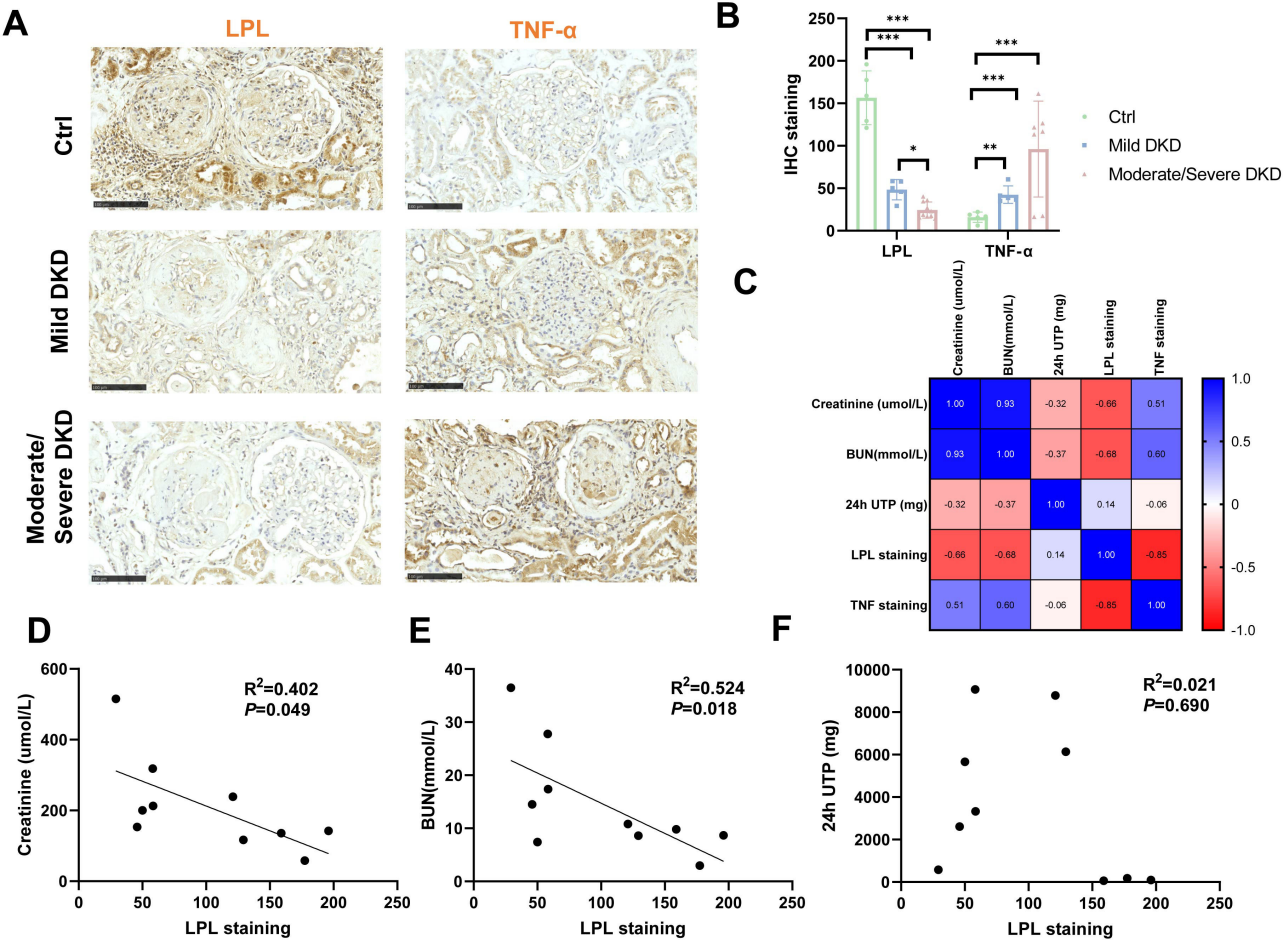
Immunohistochemical analysis of LPL and TNF-α expression in relation to DKD severity. **(A)** Representative immunohistochemical staining of LPL and TNF-α in kidney tissues from control, mild DKD, and moderate/severe DKD patients (scale bar = 100 μm). **(B)** Quantitative analysis of LPL and TNF-α staining intensities among the control, mild DKD, and moderate/severe DKD groups. *P < 0.05, **P < 0.01, ***P < 0.001. **(C)** Correlation matrix of LPL and TNF-α staining intensities with kidney function indicators (serum creatinine, urea nitrogen, and 24-hour urinary protein). **(D)** Linear regression analysis of LPL staining intensity with serum creatinine levels. **(E)** Linear regression analysis of LPL staining intensity with urea nitrogen levels. **(F)** Linear regression analysis of LPL staining intensity with 24-hour urinary protein levels.

To validate the expression pattern of LPL protein in renal tissue and its relationship with disease severity, immunohistochemistry (IHC) was performed on kidney samples from healthy controls and DKD patients with varying degrees of severity. LPL staining was reduced in DKD samples compared to controls, particularly in moderate/severe cases, while TNF-α staining appeared increased with severity (**Figure 7A**). Quantitative analysis confirmed these trends, revealing a significant decrease in LPL staining intensity with increasing DKD severity (Ctrl vs. Mild vs. Moderate/Severe DKD, *P*<0.001), whereas TNF-α staining intensity significantly increased (*P*<0.001, **Figure 7B**).

Correlation analysis was performed between LPL and TNF-α staining intensities and indicators of renal function using the combined patient and control samples (**Figure 7C**). Notably, LPL staining intensity showed a strong negative correlation with TNF-α staining intensity (r = -0.85). Furthermore, LPL staining was negatively correlated with serum creatinine (r = -0.66) and BUN (r = -0.68), while TNF-α showed positive correlations with these renal function markers. Linear regression analysis focusing on the patient samples further confirmed a significant negative correlation between LPL staining intensity and both serum creatinine (R²=0.402, *P*=0.049) (**Figure 7D**) and BUN (R²=0.524, *P*=0.018) (**Figure 7E**). While these correlations were statistically significant, the R^2^ values indicate that LPL expression explains a moderate proportion of the variance in these renal function markers. No significant correlation was found between LPL staining and 24-hour urinary protein (*P*=0.690) (**Figure 7F**). These IHC results demonstrate that LPL protein expression decreases in the kidney tissue of DKD patients as the disease progresses and is negatively correlated with renal function impairment and inflammatory response (TNF-α). This finding aligns with the LPL downregulation observed in transcriptome analyses and supports a complex role for LPL in DKD pathogenesis, potentially differing from its role in systemic lipid metabolism.

## Discussion

DKD is the leading cause of ESRD worldwide. Its incidence and medical costs continue to rise, placing a heavy burden on the medical system (21). The clinical feature of DKD is persistent albuminuria, which indicates that the disease could irreversibly progress to ESRD (22). Given the increasing global prevalence of DKD, this study identified the key role of lipoprotein metabolism-related gene LPL in DKD through systematic bioinformatic analysis, and further revealed the potential pathological mechanisms of lipid metabolism disorders and immune inflammatory responses in DKD, providing new insights and potential targets for the early diagnosis and targeted treatment of DKD.

In the progression of DKD, lipid metabolism disorders are increasingly recognized as a significant risk factor (23). Normally, the balance of lipid metabolism pathways is essential for maintaining kidney health and function (24). However, in patients with diabetes, hyperglycemia and insulin resistance lead to profound disruptions in lipid metabolism, initiating a cascade of pathological events. Excess lipids gradually accumulate in the renal tubules and glomeruli, and this abnormal lipid accumulation has been identified one of the key factors promoting the progression of DKD (25, 26). Excessive lipids not only directly damage the kidney structure, but also significantly accelerates tissue fibrosis and aggravates kidney damage by activating inflammatory responses and oxidative stress (23). Previous studies have demonstrated that the inflammatory responses caused by lipid metabolism disorders play key regulatory roles in the development of DKD. Abnormal lipid accumulation often induces the aggregation and activation of macrophages, further aggravating the inflammatory microenvironment of the kidney (27). Activated macrophages release large amounts of pro-inflammatory cytokines, such as TNF-α, IL-6, and IL-1β, which directly damage the renal tubules and glomeruli, impairing both renal structure and function (28). These cytokines also activate oxidative stress and increase the production of reactive oxygen species (ROS), accelerating renal cell apoptosis and tissue fibrosis, which ultimately promotes the progression of DKD (29). To better understand the specific mechanisms of lipid metabolism dysregulation in DKD, recent studies have utilized bioinformatic analysis and machine learning to analyze in depth gene expression data from DKD patients and healthy individuals (30). These studies suggested that lipid metabolism imbalance may influence DKD pathological process by regulating the behavior of immune cells (31, 32). It is worth noting that specific metabolic enzymes are closely related to the activity of M1 macrophages, and the increase in M1 macrophages is positively correlated with further deterioration of renal function (33, 34). In contrast, M2 macrophages have anti-inflammatory and protective effects, and their reduction may exacerbate renal damage in DKD patients (35).

As a key regulator of lipid metabolism, LPL may play a core role in lipid metabolism disorders associated with DKD. Functional enrichment analysis in this study showed that LPL genes were significantly enriched in lipid metabolism and inflammatory response pathways, especially in the Cholesterol metabolism and Glycerolipid metabolism pathways, suggesting that LPL may play an important role in the occurrence and development of DKD. In diabetic patients, due to insulin resistance and hyperglycemia, lipid metabolism is significantly disrupted, leading to lipid accumulation in the kidneys (23, 36). Previous studies have shown that abnormal lipid metabolism not only damages the glomerular and tubular structures, but also aggravates local inflammatory responses and oxidative stress, promoting the progression of DKD (23, 37). The enrichment of LPL genes in these pathways supports previous findings and further highlights its key role in regulating lipid metabolism disorders in DKD.

In addition to enrichment in lipid metabolism pathways, the significant enrichment of LPL genes in the PPAR signaling pathway also suggests that it may play an important role in regulating inflammatory responses. The PPAR pathway is a key anti-inflammatory signaling pathway that inhibits inflammatory responses by inhibiting the expression of pro-inflammatory factors (38). Previous studies have shown that activation of the PPAR signaling pathway can reduce the production of inflammatory cytokines by downregulating pro-inflammatory pathways such as NF-κB, thereby playing a protective role in chronic inflammatory diseases such as DKD (39, 40). In the immune cell infiltration analysis of this study, it was found that pro-inflammatory immune cells increased significantly in DKD patients, forming a significant pro-inflammatory immune microenvironment, which is closely related to the pathological progression of DKD. It is worth noting that the aggregation and activation of M1 macrophages are commonly accompanied by structural destruction of glomeruli and tubules (41). A previous study has shown that M1 macrophages play a crucial role in the progression of inflammation and fibrosis in DKD, and under high glucose conditions, they promote the polarization of macrophages to the M1 phenotype by downregulating STAT-3-mediated autophagy (42).

The expression level of the LPL gene was negatively correlated with M1 and M2 macrophages, suggesting that LPL may affect the pro-inflammatory microenvironment of DKD by regulating the polarization state of macrophages, thereby exacerbating kidney damage. In addition, immunohistochemistry detection revealed low LPL expression in both glomerular and tubular regions of DKD patients. This finding further supports the significant downregulation of LPL in DKD renal tissue and underscores its potential anti-inflammatory and anti-fibrotic roles in pathological progression (43). While the immunohistochemical analysis revealed a statistically significant negative correlation between LPL protein expression and renal function indicators such as serum creatinine (*P*=0.049) and BUN (*P*=0.018), it is crucial to interpret these findings in light of the relatively moderate R^2^ values (R^2^ = 0.402 for serum creatinine and R^2^ = 0.524 for BUN). These R^2^ values indicate that LPL expression explains only a moderate proportion of the observed variance in these markers. This suggests that while LPL plays a discernible contributing role in DKD progression and kidney dysfunction, its impact is part of a highly complex and multifactorial disease etiology. The pathogenesis of DKD involves intricate interactions between various factors including hemodynamic changes, metabolic disturbances, inflammation, and fibrosis. Consequently, no single gene or pathway is expected to fully account for the broad spectrum of variability observed in clinical outcomes. Therefore, while LPL has been identified as a key player in DKD, its precise impact on overall renal function needs to be understood within this broader pathological context, necessitating further studies to delineate its exact contribution and interplay with other intricate mechanisms.

In the gene-drug interaction analysis, several potential therapeutic agents that may regulate lipid metabolism and inflammatory responses through the LPL gene were identified. Among them, Ibrolipim showed a high interaction score, indicating that it may alleviate lipid metabolism disorders and related inflammatory responses in DKD by regulating LPL activity. As a lipid-lowering drug, Ibrolipim may reduce the inflammatory cascade caused by lipid accumulation by reducing the deposition of cholesterol and triglycerides in the kidneys (44). Other drugs such as anabolic steroid and acarbose also exhibited high interaction scores with LPL, suggesting potential therapeutic value in regulating LPL activity. Anabolic steroid has an immunomodulatory effect and may reduce the inflammatory damage of DKD by balancing the ratio of pro-inflammatory and anti-inflammatory immune cells; while acarbose, as a hypoglycemic agent, may indirectly regulate lipid metabolism and inflammatory responses by improving glucose metabolism (45, 46). The potential mechanisms of action of these drugs provide a variety of possible intervention pathways for the targeted treatment of DKD. By regulating the activity of LPL, these drugs are expected to not only alleviate lipid metabolism disorders but also improve the pro-inflammatory immune environment in the kidneys, reduce persistent tissue damage, and ultimately delay the progression of DKD. The significant correlation between LPL and renal function indicators (serum creatinine, urea nitrogen, and 24-hour urine protein excretion) in immunohistochemistry results further indicates that these drugs targeting LPL may have clinical application value in monitoring and improving the condition of DKD patients.

## Limitation

However, this study has certain limitations. Firstly, the sample sizes were relatively small, including only 19 DKD patients and 50 healthy controls in the training set, and 7 DKD patients and 18 healthy controls in the validation set. Additionally, the immunohistochemical experiments included only 10 kidney biopsy specimens from DKD patients with varying disease severity and 10 healthy control samples, which may not fully reflect the widespread variations in LPL expression levels among DKD patients. Future studies with larger sample sizes are needed to validate the generalizability of the results. Secondly, while this study revealed a strong association between LPL and DKD progression through bioinformatic analysis and immunohistochemistry, it is important to acknowledge that correlation does not automatically imply causation. Therefore, the exact underlying mechanisms and causal relationships still need to be further verified through *in vivo* and *in vitro* experiments to functionally validate these findings.

Furthermore, while consensus clustering identified distinct molecular subtypes of DKD, the current study, due to the inherent limitations of publicly available datasets, was unable to perform comprehensive clinical correlations with functional outcomes such as eGFR decline, which would further strengthen the clinical relevance of these subtypes. To address these limitations and establish causality, future research should focus on: (1) integrating multi-omics data analysis to reveal the complex molecular mechanisms of DKD; (2) conducting longitudinal studies to track the dynamic changes of LPL in the progression of DKD, providing stronger evidence for its role and potential as a therapeutic target; and (3) evaluating LPL-targeted therapies for their potential in precision medicine, including a focus on correlating identified molecular subtypes with specific clinical outcomes to enhance personalized diagnostic and therapeutic strategies.

## Conclusion

This study systematically identified the lipoprotein metabolism-related gene LPL as a hub gene in DKD through bioinformatic analysis and immunohistochemical validation, revealing its multiple roles in lipid metabolism disorders and immune-inflammatory response regulation. The study found that the expression level of LPL significantly decreased with the severity of DKD and was negatively correlated with the pro-inflammatory cytokine TNF-α and kidney function indicators. Further functional analyses suggested that LPL dysregulation is intricately linked to DKD progression, potentially influencing lipid metabolism and the immune microenvironment in complex ways. Additionally, gene-drug interaction analysis identified several potential drugs that may target LPL to alleviate DKD progression, providing an important theoretical basis for future clinical interventions.

## Data Availability

The original contributions presented in the study are included in the article/**Supplementary Material**. Further inquiries can be directed to the corresponding author.

## Supplementary material

Supplementary Figure 1 Gene-drug interaction analysis identifying potential therapeutic agents targeting LPL in DKD.
