# Correction: Bioinformatic analysis identifies LPL as a critical gene in diabetic kidney disease via lipoprotein metabolism

**DOI:** 10.3389/fendo.2025.1732027

**Published:** 2025-11-14

**Authors:** Qian Dong, Huan Xu, Pengjie Xu, Jiang Liu, Zhouji Shen

**Affiliations:** Department of Nephrology, The Affiliated Lihuili Hospital of Ningbo University, Ningbo, China

**Keywords:** diabetic kidney disease, lipoprotein lipase, immune cell infiltration, lipid metabolism, bioinformatic

The **Figure 5** caption were in the wrong order. The labels for panels (A) and (B) have been swapped. The order has now been corrected.

**Figure 5:**
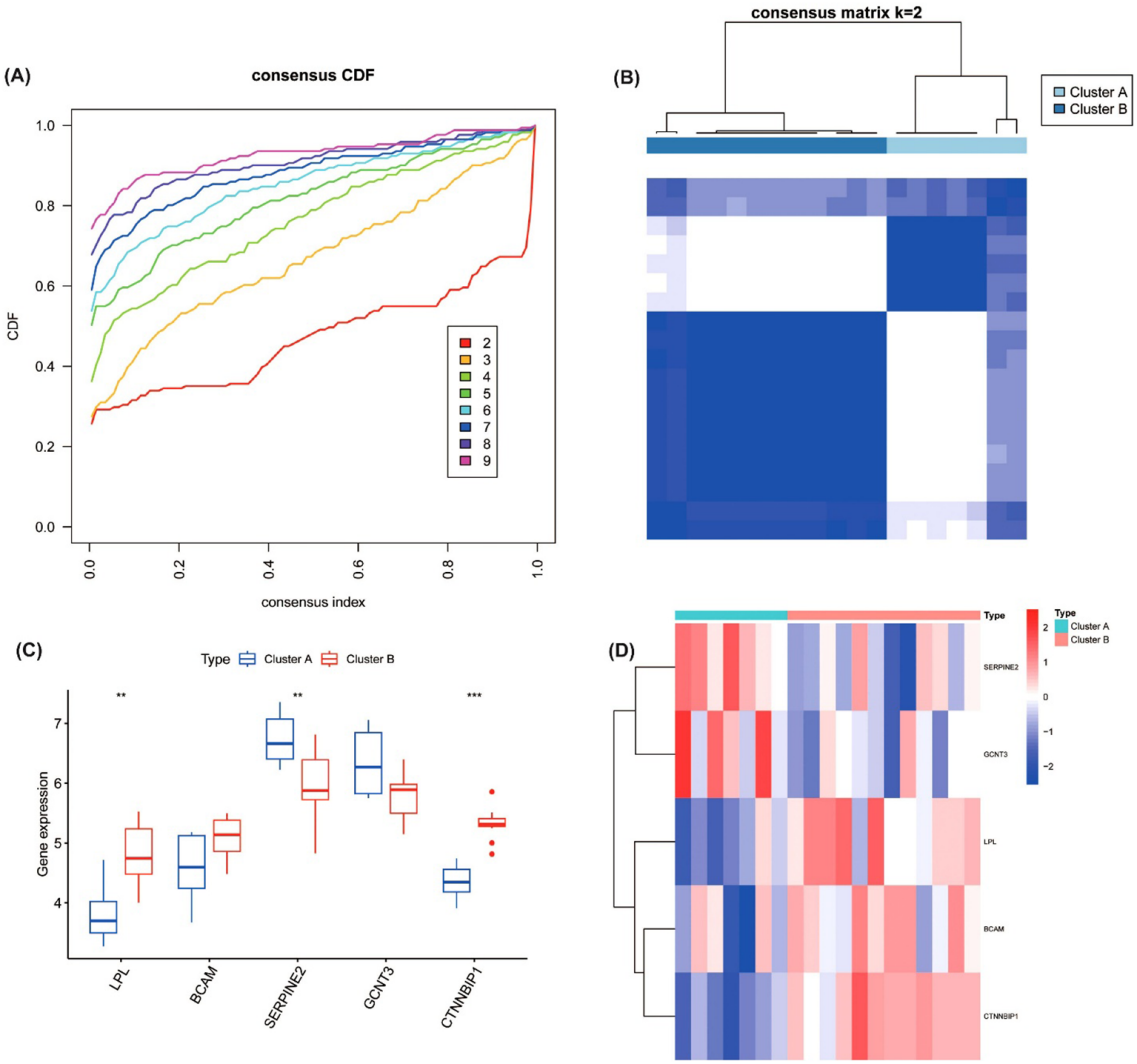
Consensus clustering analysis of five hub genes in DKD patients. **(A)** Cumulative Distribution Function (CDF) curve for consensus clustering with different values of k. **(B)** Consensus matrix for clustering, showing optimal stability at k=2, resulting in two molecular subtypes (Cluster A and Cluster B) based on the expression of hub genes. **(C)** Expression levels of the hub genes (LPL, BCAM, SERPINE2, GCNT3, and CTNNBIP1) in the two identified subtypes. ***P<0.001. **(D)** Heatmap of gene expression between Cluster A and Cluster B **P<0.01.

The error made was a directional error in the interpretation of the statistical result for the gene CTNNBIP1.

A correction has been made to the section **Results**, *Consensus cluster analysis*, Paragraph 2:

“There were significant differences in the expression levels of core genes between the two subtypes (**Figures 5C, D**). Specifically, compared to Cluster B, Cluster A exhibited significantly reduced expression of LPL (P < 0.01) and CTNNBIP1 (p < 0.001), along with lower expression of BCAM. In contrast, Cluster A showed significantly elevated expression of SERPINE2 (p < 0.01) and higher expression of GCNT3.”

The pathway names and their corresponding P-values have been corrected to match the data presented in **Table 1**.

A correction has been made to the section **Results**, *Hub gene enrichment analysis*, Paragraph 1:

“The results showed that LPL was significantly associated with Cholesterol metabolism (P = 0.012), Glycerolipid metabolism (P = 0.015) and PPAR signaling pathway (P = 0.018, **Table 1**). These findings suggest that LPL may play a crucial role in lipid metabolism disorders, and through regulating lipidmetabolism and inflammatory responses, it could contribute to the progression of DKD.”

The original version of this article has been updated.

