# Supplementary figures and images for: Bioinformatic analysis identifies LPL as a critical gene in diabetic kidney disease via lipoprotein metabolism
Source: Front Endocrinol (Lausanne). 2025 Jul 17;16:1620032. doi: 10.3389/fendo.2025.1620032 (PMC12310451; doi:10.3389/fendo.2025.1620032)

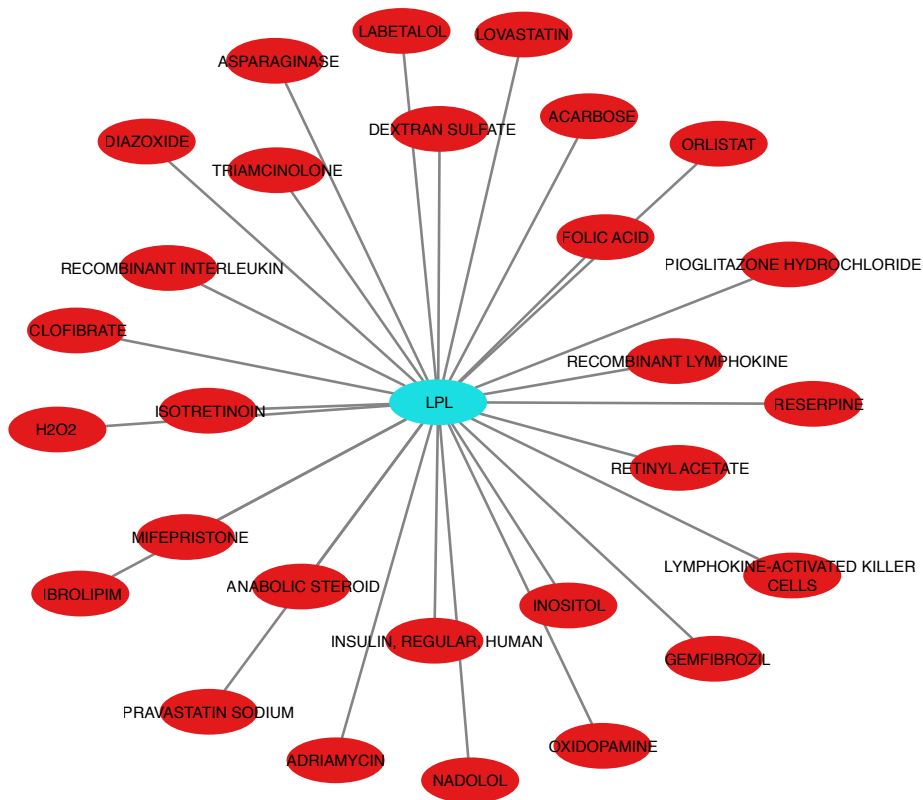

Supplement: Supplementary Figure 1 — Gene-drug interaction analysis identifying potential therapeutic agents targeting LPL in DKD. [file DataSheet1.pdf]
